# Understanding oral health care team performance in primary care: A mixed-method study

**DOI:** 10.1371/journal.pone.0217738

**Published:** 2019-05-30

**Authors:** Clarice Magalhães Rodrigues Reis, Antônio Thomaz Gonzaga Matta-Machado, João Henrique Lara Amaral, Juliana Vaz de Melo Mambrini, Marcos Azeredo Furquim Werneck, Mauro Henrique Nogueira Guimarães de Abreu

**Affiliations:** 1 Department of Community and Preventive Dentistry, Universidade Federal de Minas Gerais—UFMG, Belo Horizonte, Brazil; 2 Department of Social and Preventive Medicine, Universidade Federal de Minas Gerais, Belo Horizonte, Brazil; 3 Department of Community and Preventive Dentistry, Universidade Federal de Minas Gerais, Belo Horizonte, Brazil; 4 René Rachou Research Center, FIOCRUZ, Belo Horizonte, Minas Gerais, Brazil; University of Indianapolis, UNITED STATES

## Abstract

**Objective:**

This study aims to describe the primary care services carried out by Oral Health Teams (OHTs) in Brazil, and to understand the nuances that lead to different levels of OHT performance.

**Material & methods:**

A mixed-methods study with a sequential explanatory design was developed. In the quantitative phase, secondary data from a national survey (PMAQ-AB) was used to describe the work of 12,403 OHTs. Item response theory (IRT) was applied, to evaluate the psychometric qualities of 20 oral health questions from PMAQ-AB and to identify the performance of OHT. The quantitative results guided the selection of the qualitative sample. An extreme case sampling strategy was used (opposite results). OHTs were selected from Belo Horizonte metropolitan region in Brazil using scores measured by IRT. Data were collected through semi-structured interviews. Data analysis was conducted using deductive and inductive thematic analysis.

**Results:**

Quantitative results showed that there are OHT with high and low performance in Brazil. The IRT analysis showed that items related to prostheses and oral cancer tend to discriminate high-performance OHTs from other OHTs. Qualitative results deepened the understanding of accessing oral health services and found several access barriers, such as the insufficient number of OHTs for the population, and a very long waiting time for dental consultations other than urgency. The qualitative results confirmed that high-performance OHTs tend to emphasize oral cancer surveillance and deliver prostheses in PHC services.

**Conclusion:**

Despite the expansion of oral health in PHC in Brazil in recent years, OHTs still face many challenges such as: access barriers; failures in prevention, early diagnosis and follow-up of oral cancer cases; and insufficient rehabilitation with prostheses.

## Introduction

Evidence shows that health systems organized through primary health care (PHC) are more effective and efficient [1–3], which has led several countries to seek strategies to expand and strengthen their health systems based on the guiding principles of PHC [3]. Since 1994, Brazil has been strengthening its PHC system through the Family Health Program. In 2016, Family Health Program teams covered around 60% of the Brazilian population.

Oral health is still a challenge for Brazilians. In 2003, a national survey showed that 20% of the Brazilian population had already lost all their teeth [4]. In order to reverse this epidemiological picture, Oral Health Teams (OHTs) were included in the Family Health Strategy. The number of OHTs has grown, as has the financial incentive allocated for this purpose [5]. In 2004, the National Oral Health Policy brought expansion and qualification of oral health services, an increase in the resolution of actions, availability of removable partial and complete dentures in primary care centers, and implementation of Dental Specialty Centers (DSCs), among other activities [6]. By 2016, there were already about 33,000 OHTs in Brazil.

However, oral health in the Family Health Strategy still presents many challenges which need to be overcome in order to achieve the principles expected in PHC [2,7], including improving the quality of care provided to the population [8]. However, an integrative review confirmed the paucity of longitudinal studies assessing oral health in a PHC approach [9].

The growth and expansion of the OHT in Brazil demands evaluation of the quality of services provided. Some studies have already been conducted in order to characterize the actions and services performed by OHTs, some being local [10,11], and others carried out at the national level with quantitative methods [12,13]. In Brazil, OHT are composed by one dentist and one dental assistant (OHT type I) or one dentist, one dental assistant and one dental hygienist (OHT type II). OHT provides community and clinical procedures, such as surgical, restorative and preventive procedures and dental emergencies at PHC level. Evaluation research has been conducted, integrating quantitative and qualitative methods in order to enhance research, since the use of these combined methods promotes a better understanding of the problems studied than the isolated use of one of the approaches [14]. However, its application in the evaluation of oral health services has been little identified in the scientific literature.

Thus, this study aims to describe the PHC actions carried out by OHTs in Brazil, and to understand aspects that lead to different levels of OHT performance.

## Methods

### Ethics statement

The study was submitted to and approved by the Brazilian Ethics Committee and by the Ethics Committee for Human Research of the Universidade Federal de Minas Gerais (protocol numbers CAAE 02396512.8.0000.5149 and 31525514.9.0000.5149). Individuals signed the informed consent, the participation was voluntary and they could refuse to answer any part of the questions. Publicly available, de-identified data from the Brazilian Ministry of Health were also analyzed.

### Study design

This is an explanatory mixed-method research. The mixed explanatory design is a study of two distinct and sequential phases, in which the quantitative phase is followed by a qualitative phase. The researcher first collects and analyzes quantitative data and then collects and analyzes qualitative data that will help explain the results found in the quantitative phase [14]. That is, the second (qualitative) phase is built from the first phase and the two phases are connected in the intermediate and final stages of the research. The justification for this approach is that the quantitative phase promotes a general understanding of the research problem studied, while the qualitative phase refines and deepens the statistical results by exploring the vision of the social actors involved, contributing to clarify the gaps in the previous phase [15].

In this case, the quantitative phase was developed from analyzing secondary data collected by the Brazilian Ministry of Health as part of an evaluation pay for performance program. This dataset was used to describe the work of 12,403 OHTs and Item response theory (IRT) [16] was applied to develop a construct called “OHT performance”. This quantitative phase showed initial findings that were explored further in a qualitative phase. Thus, we integrated this mixed method study in two moments: 1) selecting participants for a qualitative phase by choosing a purposefully extreme case sampling, i.e., OHTs from Belo Horizonte metropolitan region placed in the extreme quintiles according to the IRT scores, 2) discussing the results by mixing the quantitative findings and qualitative findings. This gave us a better phenomenon understanding, which was the performance of OHT.

The flowchart of this mixed methodology is illustrated in S1 Fig.

### Quantitative phase

This first research phase used secondary data from a national survey performed by the Brazilian Ministry of Health in 2012, i.e., the Program for Improving Access and Quality of Primary Care (PMAQ-AB). PMAQ-AB is a performance payment program evaluation aimed to improve the quality of PHC services in Brazil, including oral health care services [17].

Data for 12,403 OHTs which answered the questionnaire were used. IRT [16] was applied, to evaluate the psychometric properties of the oral health questions used in the PMAQ-AB questionnaire [13]. The IRT model relates the likelihood of an individual’s response to an item and its latent trait (or construct). A latent construct is a characteristic that cannot be measured directly, such as attitude, satisfaction or proficiency, that is, a latent, unobservable variable that will be estimated based on the responses given to each of the items considered by the respondents participating in the study [17,18]. In this study, the latent construct derived from the application of IRT was the performance of OHTs, measured from 20 questions about dental procedures performed in primary care (Table 1) [19]. The IRT model provided a score, a rating grade, for each OHT according to the responses given to the PMAQ-AB questionnaire, called OHT performance (provision of primary dental care procedures) This score could theoretically vary from -4 (OHT with lowest performance) to +4 (OHT with best performance). Each oral health team received a score that can range from lowest performance (lowest frequency of primary dental care procedures) to highest performance (highest frequency of primary dental care procedures). More details have been published previously [13].

**Table 1:** Questions about dental procedures in PHC used for creating a rating grade–OHT performance.

Did your OHT (perform)?:
Patient welcoming
Disease risk classification at the first appointment
Oral health services according to a patient’s risk
Guidelines for “patient welcoming” with documentation
Ensure continuity of care with documentation
References for prosthetics services
Sealants
Fluoride application
Amalgam filling
Composite filling
Dental extraction
Temporary restorations
Endodontic medication use in emergencies
Drainage of oral abscesses
Supragingival scaling, root planing and coronal polishing
Denture impressions with documentation
Provide the dentures and follow the patients with documentation
Identify oral lesions and referring suspected cases of oral cancer
Register and follow cases of oral cancer with documentation
Identifying people who need dentures

### Qualitative phase

The objective of this phase was to understand aspects that lead to the different levels of performance of OHTs identified in the previous phase [20]. Integration of this mixed-methods study happened during participant selection for qualitative interviews: the quantitative results guided the selection of the qualitative sample. An extreme case sampling strategy was used (opposite results) [21]. OHTs were selected from scores measured by IRT, which originates the “OHT performance” construct. We chose to sampling OHT from the metropolitan region of Belo Horizonte. The metropolitan region of Belo Horizonte, with around 6 million people, was composed by 33 municipalities (Baldim, Betim, Brumadinho, Caeté, Capim Branco, Confins, Contagem, Esmeraldas, Florestal, Ibirité, Igarapé, Itaquara, Itatiaiuçu, Jaboticatubas, Juatuba, Lagoa Santa, Mário Campos, Mateus Leme, Matozinhos, Nova Lima, Nova União, Pedro Leopoldo, Raposos, Ribeirão das Neves, Rio Acima, Rio Manso, Sabará, Santa Luzia, São Joaquim de Bicas, São José da Lapa, Sarzedo, Taquaraçu de Lima, Vespasiano) cities and towns with a diverse socioeconomic development levels. Thus, IRT scores from all OHT that participated in PMAQ-AB in those 33 cities (n = 65) were displayed in a range from lowest to highest performance. All OHTs in the extreme quintiles (less than −0.40 and greater than 0.70) that had maintained the same dentist since quantitative data collection were included (n = 10).

Data were collected through semi-structured interviews. An interview guide was applied that contained interviewee’s demographics, and questions about access to oral health services, integrality of actions and coordination of care, especially with regard to work with oral cancer prevention and prosthesis supply in PHC. This interview guide was piloted before fieldwork in two interviews tests in order to adjusting and refining questions and probes. The average duration of the interview was 30 minutes. The interviews were recorded with the permission of the participants, and were transcribed verbatim.

The demographic data of the professionals interviewed in the metropolitan region of Belo Horizonte (n = 10) and the performance of their respective OHTs are shown in Table 2. All OHT were due to provide community and clinical procedures, such as surgical, restorative and preventive procedures and dental emergencies at PHC level.

**Table 2:** Demographic and health service data of interviewed’ dentists.

	Performance IRT	Score IRT	Sex	Age	Hired for contract	Service time (years)	Type of OHT*	Population served^†^
T1H	high	0.79	F	62	permanent	20	Type II	3,890
T2H	high	0.79	F	54	temporary	3	Type I	2,000
T3L	low	-0.41	F	47	permanent	3	Type II	3,486
T4L	low	-0.48	F	32	permanent	6	Type I	3,750
T5L	low	-0.55	M	26	temporary	3	Type II	2,444
T6H	high	1.09	F	37	permanent	3	Type II	3,617
T7H	high	0.90	M	33	permanent	3	Type I	2,180
T8H	high	0.76	F	29	permanent	2	Type I	1,976
T9L	low	-0.46	F	28	temporary	2	Type II	4,570
T10L	low	-2.01	M	54	temporary	3	Type I	4,300

* OHT type I–one dentist and one dental assistant; OHT type II–one dentist, one dental assistant and one dental hygienist.

† Source: Ministry of Health. Brasil. Saúde mais perto de você - acesso e qualidade—Programa Nacional de Melhoria do Acesso e da Qualidade da Atenção Básica (PMAQ)—dados. 2012. Available from: http://dab.saude.gov.br/portaldab/ape_pmaq.php?conteudo=1_ciclo

Data analysis was conducted using deductive and inductive thematic analysis proposed by de Braun and Clarke [22]. Two authors (CR and MHA) coded those initial two interviews to check and adjust the initial codes in order to reflect upon the initial results [23]. Then, one of the authors (CR) immersed herself in the data analysis in an inductive way, seeking what emerged most from the interviewees’ speech After this first analysis, a second coding of the interviews happened in a deductive way, keeping in mind the theoretical reference of PHC [2] attributes and the objective of the qualitative phase of this mixed project, which was to respond in such a way that the qualitative results deepen the quantitative results.

## Results

Despite the theoretical scores could varied from -4 to +4, in this study the scores of OHT performance for all Brazilian OHTs surveyed (n = 12,403) varied from -2.76 to +2.12 (S2 Fig). IRT analysis also shows that OHTs with high performance develop actions to identify oral lesions and referring suspected cases of oral cancer (80.4%) and provide the dentures and follow the patients with documentation (30.1%) more frequently than lowest scores OHTs. So, these two questions had high difficulty parameter value by IRT.

Four main themes emerged from the qualitative data: access barriers, continuity of care, actions to combat oral cancer, and total prosthesis in PHC.

### Barriers to accessing oral health services in PHC

Challenges to accessing oral health services in PHC emerged in the interviews. It was observed in all OHTs, regardless of the level of performance, that there is a separation between users who access the service with urgent dental needs (they need immediate care, usually pain relief), and those who come with other complaints, whose consultation can be scheduled for another time. High-performance OHTs reported strategies to schedule treatment for users who come with urgent needs.

“I always catch him on the urgency treatment because it’s very rare I do not convince him to return to the treatment.” (T2H)

On the other hand, low-performance OHTs use other forms of access to dental treatment, such as waiting lists and telephone consultations. In some cases, patients wait up to 7 months to be called for care; in other cases, this appointment happens after years of waiting.

“It takes about 6, 7 years for us to call people in the wait list. And we are having a lot of troubles, because sometimes the telephone number changes and there is no contact with the patient.” (T5L)

In one case, the professional reported performing a risk classification as a way of organizing the first dental appointment.

“It has welcoming services every Tuesday. People come, we already look and sort by code 0, 1, 2, 3, 4, 5 and put the name in the notebook. There is a notebook for each code.” (T9L)

However, when the dentist was asked if he uses this risk classification criterion to organize the appointment and prioritization, the answer was negative. The risk classification is done because it is required by the municipality.

Welcoming services have been reported differently, and are often misunderstood as user triage in both high-performance and low-performing OHTs.

“We always make the welcome service on Tuesday. People come, get the examination, the classification, and their name goes in the notebook.” (T1H)

“The person arrives at the reception, the girl evaluates how many teeth she has to deal with, places her name in the notebook and the person waits for the vacancy.” (T9L)

Understanding of the welcoming service was also diverse. Some understand it as part of user triage, others as part of humanization strategies, and others as a way to involve all members of the PHC center in providing good information and welcoming users.

Another barrier to access was the exacerbated number of users for each OHT. This was a constant complaint in low-performing OHTs. This reality causes tensions between health staff and users.

### Continuity of care

Another theme that emerged from interviews was longitudinal care. OHTs (high and low performance) reported that scheduling return appointments is used to track their users and prevent new oral diseases. Following users is seen as a way to prevent future oral problems.

“There’s a notebook. I’m going to write down, and every 6 months I call the patient back, which for me this return is sacred, and it works. I was surprised because very quickly I was able to control the oral health of the people here.” (T2H)

A high-performance team reported working with care throughout the life cycles, creating a link between professional and patient. It started following pregnant women and their babies.

“The baby is very easy, they come. I have treated a lot of mothers, so babies really come, it is spontaneous demand.” (T6H)

### Actions to oral cancer care

In general, prevention and early detection of oral cancer are emphasized by high- and low-performance OHTs during campaigns to vaccinate the elderly against influenza. High-performance OHTs reported talking about oral cancer prevention throughout the year in the primary care center waiting room and in community centers.

“Two to three times a year we talk about oral cancer in the community centers. The primary care center reception has posters on oral cancer, and we teach self-examination.” (T8H)

In general, the network of services for monitoring suspected cases of oral cancer, which involves consultation with a specialist for biopsy, is deficient. The reference service for secondary care, in many cases, does not exist in municipalities. In the few municipalities that have the reference service, the OHT only directs the patient, and the specialist proceeds to monitor cases in isolation.

“Here, I used to follow the patients before, make this control … but now with the CEO (reference center), the stomatologist is requiring follow-up of those patients.” (T4L)

In high-performance OHTs, even when the municipality does not provide secondary care in the field of stomatology, dentists use their personal network to follow up on suspected cases of oral cancer.

“Especially edentates, I check to see those in question. If I suspect, I’ll send them to PUC (“Pontifical Catholic University of Minas Gerais”).” (T6H)

“… as I have connections in Belo Horizonte, I already direct them to Odilon Behrens. It’s informal, it’s just because I know it. I have acquaintances in Belo Horizonte.” (T7H)

### Prosthetics in PHC

The provision of prostheses to patients in PHC is concentrated in high-performance OHTs.

“Every dentist makes his prosthesis as soon as he finishes the treatment. He goes to the social worker, he takes the referral he needs for the income.” (T6H)

In a high-performance OHT, the dentist provided a prosthetic service in a rural area, avoiding displacement of patients for long distances.

“Before everything went to the DSC, I do not know if it is because the dentist did not perform them, so now, as the material comes here, I will continue to make them here, so I do not need to be moving the patient to headquarters.” (T2H)

However, in another OHT, prosthesis provision does not happen in PHC, but in the DSC or other reference service of the municipality. However, in some municipalities, the prosthesis provision does not exist.

“It has a very large demand, but it still does not have the service. We feel bad, you do the treatment, the mouth is ready for the prosthesis, but then the patient comes back at the next appointment in 6 months, 1 year, and he’s still without anything, he’s toothless still.” (T3L)

## Discussion

This mixed-methods study showed that most OHTs perform the expected dental procedures in PHC. Procedures related to prostheses and the prevention of oral cancer are performed by a much smaller number of OHTs usually with higher performance.

The importance of facilitating access to PHC services is extensively documented, emphasizing PHC’s leading role as a “gateway” to the service. Initially, in the quantitative phase, the findings of this study pointed to a better-structured service network, in which most OHTs report perform primary care procedures. However, the qualitative analysis deepened these findings, and demonstrates several barriers to accessing oral health services via OHTs. One example is the insufficient number of OHTs for the population. This was a complaint in low-performance OHT interviews. The recommendation of the Ministry of Health is to have one OHT for a maximum of 4,000 inhabitants [24]. During the interviews, there were cases identified where an OHT was a reference for 20,000 inhabitants. Official data (Table 2) has shown that the population served is lower than this number of inhabitants said by one dentist. However the number of population served is still sometimes higher than the recommended by the Ministry of Health. In those cases, although the physical structure is considered adequate, the allocation of human resources is insufficient for the population needs, generating exclusion.

Another barrier to access found was the way of scheduling appointments. Many OHTs still have a time and date set for scheduling appointments, which is an access barrier [13]. Qualitative interviews added the problem of waiting time to access these dental appointments, which can reach 7 months in some cases. This large space between scheduling and consultation can lead to forgetfulness, which has already been described as one reason for a lack of dental consultation [25,26]. That is, this form of demand organization becomes extremely inefficient since, besides setting up access barriers, it generates future absenteeism and team idleness.

It has also been found that OHTs that perform risk classification do not use it for prioritizing scheduling appointments, turning it into a bureaucratic function. Dental classification and risk assessment for prevention of dental morbidity have emerged as alternatives to setting appointment priorities because they are simpler to perform than epidemiological surveys [27,28]. This system is criticized for reinforcing the biomedical model to guide prioritization for scheduling appointments [28]. This research found that OHTs spend time performing risk classification that is not used. Municipal health managers should reflect upon required mandates that could be useless and tiresome for health professionals.

Still on access, the majority of OHTs reported performing a welcome service, but no protocols followed were presented [13]. Qualitative data, to a certain extent, confirm this information. OHTs presented a variety of levels of understanding about a welcome service, which is often confused with user triage for organization of the service. Although most OHTs claim to perform a welcome service, non-use of protocols seems to facilitate confusion between a welcome service and user triage, as found in other studies [29].

Longitudinal oral health care includes, among other actions, access to dental treatment, as well as periodic return visits that allow the user to maintain a good oral health condition [30]. Quantitative results did not capture that nuance, which was added to the qualitative analysis when only high-performance OHTs answered this question. Those teams demonstrated a more advanced level of organization to follow patients throughout their life, prioritizing return visits and, in some cases, following users since their mother’s pregnancy.

Dealing with oral cancer in terms of prevention, screening and rehabilitation was fundamental to distinguish between high- and low-performance OHTs. In the quantitative phase, OHTs that carry out actions to deal with oral cancer demonstrated a higher level of performance, which was confirmed in the qualitative interviews. In Brazil, PHC centers usually plan health promotion actions while vaccination campaigns happen. Thus, during influenza vaccination campaigns for the elderly, OHTs take the opportunity to do mouth screening in the population to look for early signs of oral cancer. The practice of oral cancer screening in the elderly is the most common prevention service provided by OHTs. However, a recent systematic review concluded that there is insufficient scientific evidence to prove that this screening method reduces the mortality rate from oral cancer [31]. Another study showed that visual screening for early detection of oral cancer appears to be more helpful in reducing mortality rates in alcohol and tobacco users. The combined use of alcohol and tobacco is considered a main risk factor for oral cancer, being linked to more than 80% of cases [32]. Thus, it is important that OHTs target these populations for screening instead of the elderly.

Qualitative results also clarified that access to a specialized stomatology service is a challenge to high- and low-performance OHTs. However, high-performance teams tend to rely on dentists’ personal contacts to refer patients, often using their personal contacts to refer patients to the stomatology service when a biopsy is needed, and to follow those patients. Altruism has been recognized as a desirable attitude among health professionals, which should be encouraged even in training [33,34]. Burks and Youll [35] demonstrated that empathy and altruism are related, and that involvement with patients is an important attitude among health professionals, with the aim of producing more efficient results and greater user satisfaction.

A small number of OHTs at the national level perform total prosthesis in PHC centers. This information was essential to classify OHTs as low or high performance [13]. Higher-performance teams tend to offer a prosthetic service, ensuring user rehabilitation. This finding was reinforced in the qualitative phase. Tooth loss in Brazil is still considered very high, especially in poor regions [36] where the Family Health Program is well spread. Therefore, it is important to deliver prostheses in those settings, for rehabilitation of the population and to improve quality of life [37,38].

This mixed method study integrated quantitative and qualitative phase in two moments: when quantitative results, i.e. OHT score performance were used to guide the qualitative sample selection, and again to explain the research results, i.e., qualitative results emphasize quantitative findings that linked oral cancer surveillance and prostheses deliver to OHT higher performance.

Some individual characteristics of professionals, such as altruism [33–35] and other structural issues in health services, such as work health conditions [13,25,26] were clearly different among OHTs with different IRT scores. Considering that these scores measure provision of primary dental care procedures, an attitudinal issue of these OHTs, public policies could focus on factors that could enhance their attitudes. A balance between the training of the dental team in an appropriate way to the needs of population and the guarantee of satisfactory conditions of work may be helpful [39]. This study has limitations. The quantitative phase was a cross-sectional descriptive study with low analytical power. However, these findings were important to map OHT actions in Brazil. Another limitation is the methodology of the PMAQ-AB program, from which the quantitative data were extracted. Because it is a pay-for-performance program, there may have been information bias with a tendency toward positive responses. Yet, for this first evaluation of the PMAQ-AB in 2002, the Ministry of Health mandated that only 50% of OHTs in each municipality could enroll in PMAQ. This might have added selection bias due to a tendency to select more structured OHTs, since it is a performance payment program. However, those issues have been clarified in the qualitative phase as high and low performances OHT were interviewed. One limitation of the qualitative phase is that the data were reduced to the metropolitan region of Belo Horizonte. Despite the metropolitan region of Belo Horizonte did not statistically represent all Brazilian cities and towns, its socioeconomic diversity is quite similar to Brazilian scenario. Moreover it was possible to clarify many points of the previous quantitative phase, demonstrating the potential of mixed methodology for the diagnosis of health services.

## Conclusion

The results of this mixed-methods research show that, despite the expansion of oral health in PHC in Brazil in recent years, OHTs still face many challenges such as: access barriers; failures in prevention, early diagnosis and follow-up of oral cancer cases; and insufficient rehabilitation with prostheses. Given the importance of that health condition, public policies should continue addressing oral health care demand. Thus, there is a need to attempt to deliver PHC principles in oral care.

## Supporting information

S1 Fig Visualization of the explanatory mixed study of two sequential phases Source: Adapted from Ivankova. 200615. (TIF)

S2 Fig Scores of Oral Health Teams performance, by item response theory, 2012. (TIF)
