# Supplementary figures and images for: Understanding oral health care team performance in primary care: A mixed-method study
Source: PLoS One. 2019 May 30;14(5):e0217738. doi: 10.1371/journal.pone.0217738 (PMC6542530; doi:10.1371/journal.pone.0217738)

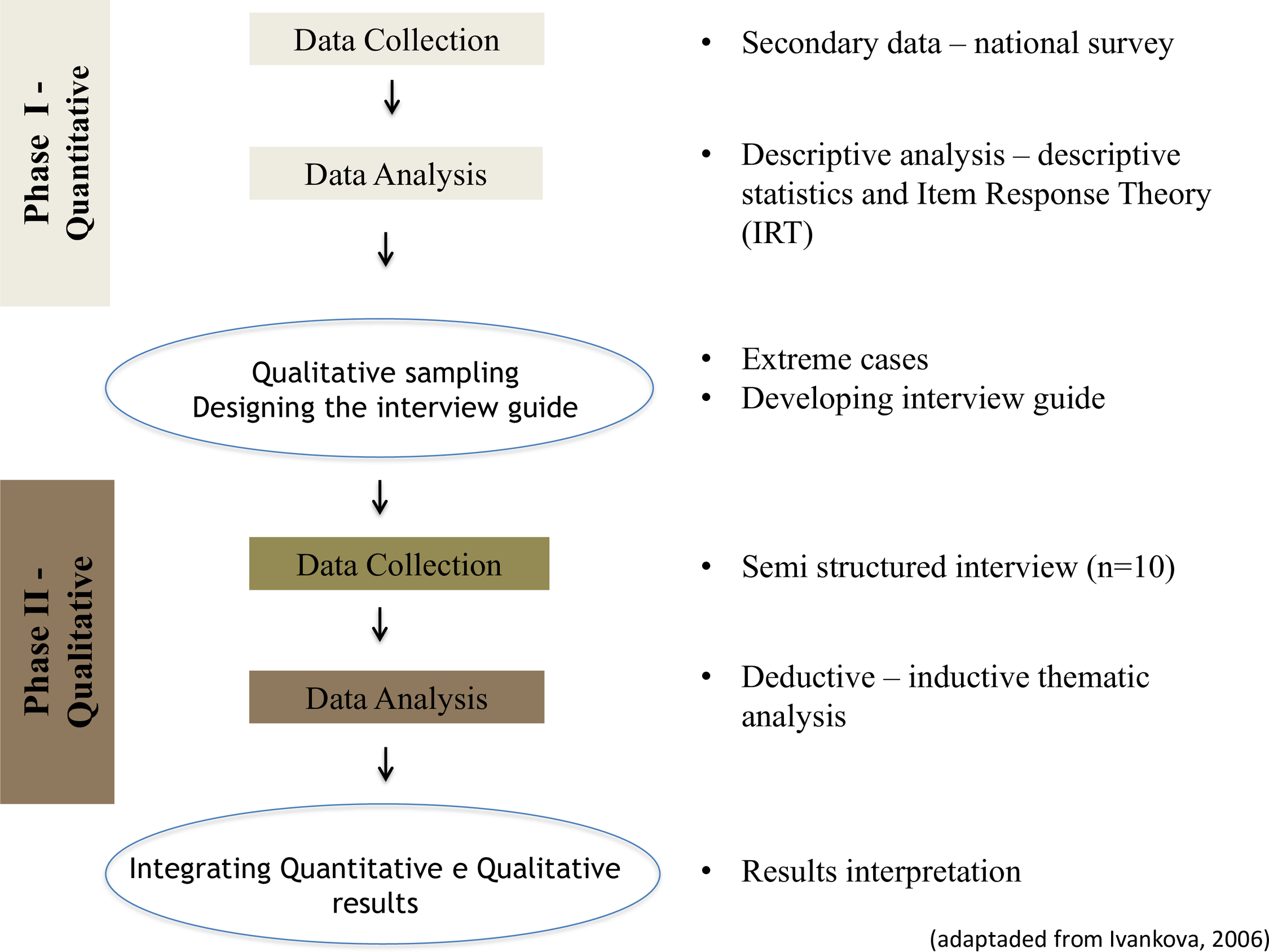

Supplement: S1 Fig — (TIF) [file pone.0217738.s001.tif]

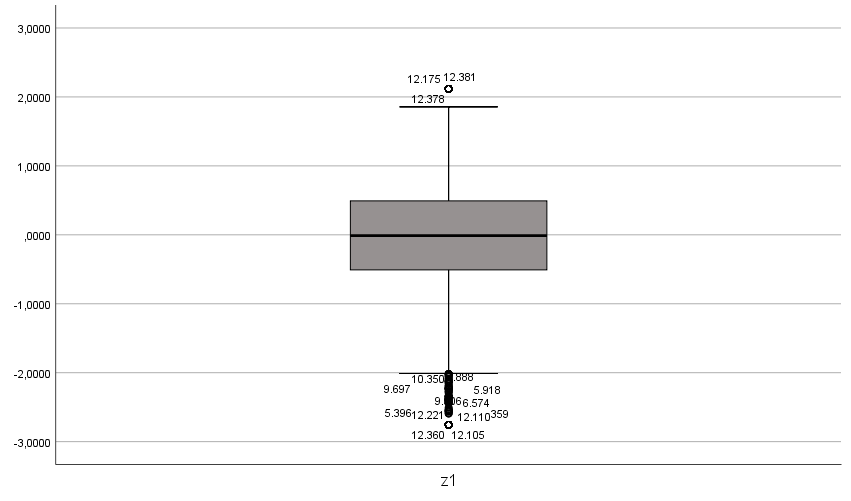

Supplement: S2 Fig — (TIF) [file pone.0217738.s002.tif]
